# Supplementary material for: Modeling glioblastoma heterogeneity as a dynamic network of cell states
Source: Mol Syst Biol. 2021 Sep 16;17(9):e10105. doi: 10.15252/msb.202010105 (PMC8444284; doi:10.15252/msb.202010105)
Supplement: Supplementary file 5 — Source Data for Figure 3 [file MSB-17-e10105-s001.zip › Figure3A_sourcedata/GSEA_3065/hallmarks_state2.GseaPreranked.1623416269049/HALLMARK_COMPLEMENT.html]

Details for gene set HALLMARK\_COMPLEMENT[GSEA]

|  || Dataset | state2 |
| Phenotype | NoPhenotypeAvailable |
| Upregulated in class | na\_neg |
| GeneSet | HALLMARK\_COMPLEMENT |
| Enrichment Score (ES) | -0.5342955 |
| Normalized Enrichment Score (NES) | -2.0556636 |
| Nominal p-value | 0.0 |
| FDR q-value | 0.0 |
| FWER p-Value | 0.0 |
Table: GSEA Results Summary

  

Fig 1: Enrichment plot: HALLMARK\_COMPLEMENT      
 Profile of the Running ES Score & Positions of GeneSet Members on the Rank Ordered List

  

| PROBE | GENE SYMBOL | GENE\_TITLE | RANK IN GENE LIST | RANK METRIC SCORE | RUNNING ES | CORE ENRICHMENT || 1 | PFN1 |  |  | 352 | 0.216 | -0.0157 | No |
| 2 | CALM1 |  |  | 409 | 0.198 | -0.0016 | No |
| 3 | GNB2 |  |  | 432 | 0.193 | 0.0156 | No |
| 4 | CALM3 |  |  | 446 | 0.191 | 0.0336 | No |
| 5 | KIF2A |  |  | 633 | 0.158 | 0.0297 | No |
| 6 | PPP4C |  |  | 928 | 0.125 | 0.0110 | No |
| 7 | USP14 |  |  | 1101 | 0.112 | 0.0039 | No |
| 8 | FDX1 |  |  | 1224 | 0.103 | 0.0013 | No |
| 9 | ATOX1 |  |  | 1246 | 0.102 | 0.0094 | No |
| 10 | USP16 |  |  | 1318 | 0.097 | 0.0116 | No |
| 11 | DUSP6 |  |  | 1345 | 0.095 | 0.0185 | No |
| 12 | PPP2CB |  |  | 1355 | 0.095 | 0.0271 | No |
| 13 | GNB4 |  |  | 1403 | 0.092 | 0.0315 | No |
| 14 | PLSCR1 |  |  | 1853 | 0.070 | -0.0095 | No |
| 15 | MSRB1 |  |  | 1928 | 0.067 | -0.0106 | No |
| 16 | RHOG |  |  | 1944 | 0.067 | -0.0054 | No |
| 17 | ZEB1 |  |  | 1995 | 0.065 | -0.0041 | No |
| 18 | CASP3 |  |  | 2028 | 0.064 | -0.0011 | No |
| 19 | RNF4 |  |  | 2065 | 0.062 | 0.0013 | No |
| 20 | PSMB9 |  |  | 2093 | 0.061 | 0.0047 | No |
| 21 | GPD2 |  |  | 2101 | 0.061 | 0.0101 | No |
| 22 | CA2 |  |  | 2111 | 0.061 | 0.0153 | No |
| 23 | RABIF |  |  | 2395 | 0.051 | -0.0098 | No |
| 24 | RBSN |  |  | 2535 | 0.047 | -0.0199 | No |
| 25 | MT3 |  |  | 2560 | 0.046 | -0.0178 | No |
| 26 | LAP3 |  |  | 2647 | 0.043 | -0.0227 | No |
| 27 | ANXA5 |  |  | 2812 | 0.039 | -0.0363 | No |
| 28 | GMFB |  |  | 2842 | 0.038 | -0.0356 | No |
| 29 | CASP7 |  |  | 3134 | 0.031 | -0.0635 | No |
| 30 | TFPI2 |  |  | 3215 | 0.029 | -0.0691 | No |
| 31 | GNAI3 |  |  | 3356 | 0.027 | -0.0814 | No |
| 32 | GNG2 |  |  | 3367 | 0.026 | -0.0798 | No |
| 33 | RAF1 |  |  | 3447 | 0.025 | -0.0857 | No |
| 34 | GCA |  |  | 3565 | 0.023 | -0.0959 | No |
| 35 | SIRT6 |  |  | 3657 | 0.021 | -0.1035 | No |
| 36 | GRB2 |  |  | 3991 | 0.016 | -0.1376 | No |
| 37 | GNAI2 |  |  | 4037 | 0.015 | -0.1409 | No |
| 38 | SH2B3 |  |  | 4182 | 0.012 | -0.1550 | No |
| 39 | PRDM4 |  |  | 4313 | 0.010 | -0.1679 | No |
| 40 | IRF2 |  |  | 4326 | 0.010 | -0.1682 | No |
| 41 | LTA4H |  |  | 4370 | 0.009 | -0.1718 | No |
| 42 | XPNPEP1 |  |  | 4637 | 0.005 | -0.1997 | No |
| 43 | DGKH |  |  | 4858 | 0.002 | -0.2231 | No |
| 44 | BRPF3 |  |  | 4960 | 0.000 | -0.2339 | No |
| 45 | EHD1 |  |  | 5018 | -0.000 | -0.2399 | No |
| 46 | PIK3CA |  |  | 5084 | -0.001 | -0.2468 | No |
| 47 | VCPIP1 |  |  | 5279 | -0.005 | -0.2671 | No |
| 48 | DYRK2 |  |  | 5426 | -0.007 | -0.2819 | No |
| 49 | CEBPB |  |  | 5541 | -0.009 | -0.2932 | No |
| 50 | RCE1 |  |  | 5566 | -0.009 | -0.2948 | No |
| 51 | USP15 |  |  | 5812 | -0.013 | -0.3197 | No |
| 52 | CSRP1 |  |  | 6030 | -0.017 | -0.3412 | No |
| 53 | USP8 |  |  | 6074 | -0.018 | -0.3440 | No |
| 54 | FYN |  |  | 6106 | -0.018 | -0.3454 | No |
| 55 | AKAP10 |  |  | 6288 | -0.021 | -0.3626 | No |
| 56 | LGMN |  |  | 6457 | -0.025 | -0.3781 | No |
| 57 | STX4 |  |  | 6663 | -0.029 | -0.3971 | No |
| 58 | IRF7 |  |  | 6755 | -0.031 | -0.4037 | No |
| 59 | PLAUR |  |  | 6906 | -0.034 | -0.4163 | No |
| 60 | CTSO |  |  | 7105 | -0.039 | -0.4335 | No |
| 61 | L3MBTL4 |  |  | 7119 | -0.040 | -0.4309 | No |
| 62 | PREP |  |  | 7295 | -0.045 | -0.4451 | No |
| 63 | CPQ |  |  | 7468 | -0.050 | -0.4584 | No |
| 64 | IRF1 |  |  | 7563 | -0.052 | -0.4632 | No |
| 65 | F3 |  |  | 7662 | -0.055 | -0.4681 | No |
| 66 | PCLO |  |  | 7696 | -0.056 | -0.4659 | No |
| 67 | PSEN1 |  |  | 7761 | -0.058 | -0.4669 | No |
| 68 | LIPA |  |  | 8178 | -0.076 | -0.5036 | No |
| 69 | DOCK10 |  |  | 8315 | -0.084 | -0.5097 | No |
| 70 | MMP15 |  |  | 8489 | -0.095 | -0.5186 | No |
| 71 | CTSL |  |  | 8637 | -0.106 | -0.5236 | Yes |
| 72 | C1R |  |  | 8644 | -0.107 | -0.5134 | Yes |
| 73 | CPM |  |  | 8835 | -0.127 | -0.5208 | Yes |
| 74 | S100A13 |  |  | 8880 | -0.133 | -0.5120 | Yes |
| 75 | COL4A2 |  |  | 9069 | -0.161 | -0.5158 | Yes |
| 76 | PDP1 |  |  | 9089 | -0.164 | -0.5013 | Yes |
| 77 | PLAT |  |  | 9145 | -0.176 | -0.4892 | Yes |
| 78 | PRCP |  |  | 9153 | -0.178 | -0.4719 | Yes |
| 79 | CTSB |  |  | 9255 | -0.215 | -0.4609 | Yes |
| 80 | ADAM9 |  |  | 9285 | -0.226 | -0.4411 | Yes |
| 81 | CD46 |  |  | 9300 | -0.230 | -0.4193 | Yes |
| 82 | LGALS3 |  |  | 9308 | -0.236 | -0.3961 | Yes |
| 83 | CTSD |  |  | 9320 | -0.246 | -0.3722 | Yes |
| 84 | LRP1 |  |  | 9338 | -0.259 | -0.3477 | Yes |
| 85 | CD59 |  |  | 9358 | -0.283 | -0.3210 | Yes |
| 86 | FN1 |  |  | 9364 | -0.294 | -0.2916 | Yes |
| 87 | TIMP2 |  |  | 9379 | -0.316 | -0.2611 | Yes |
| 88 | LAMP2 |  |  | 9380 | -0.322 | -0.2284 | Yes |
| 89 | TIMP1 |  |  | 9387 | -0.334 | -0.1951 | Yes |
| 90 | HSPA5 |  |  | 9406 | -0.395 | -0.1569 | Yes |
| 91 | CTSC |  |  | 9413 | -0.431 | -0.1138 | Yes |
| 92 | CLU |  |  | 9430 | -0.547 | -0.0600 | Yes |
| 93 | CTSV |  |  | 9434 | -0.601 | 0.0006 | Yes |
Table: GSEA details [plain text format]

  

Fig 2: HALLMARK\_COMPLEMENT: Random ES distribution      
 Gene set null distribution of ES for **HALLMARK\_COMPLEMENT**
